# The impact of pre-freezing storage time and temperature on gene expression of blood collected in EDTA tubes

**DOI:** 10.1007/s11033-022-07320-5

**Published:** 2022-03-12

**Authors:** Serena Martire, Paola Valentino, Fabiana Marnetto, Luca Mirabile, Marco Capobianco, Antonio Bertolotto

**Affiliations:** 1 Clinical Neurobiology Unit, Neuroscience Institute Cavalieri Ottolenghi (NICO), Regione Gonzole 10, 10043 Orbassano, Italy; 2 Department of Neuroscience “Rita Levi Montalcini”, University of Turin, Via Cherasco 15, 10100 Turin, Italy; 3 SCDO Neurologia and CRESM, University Hospital AOU San Luigi Gonzaga, Regione Gonzole 10, 10043 Orbassano, Italy; 4 Koelliker Hospital, 10100 Turin, Italy

**Keywords:** EDTA tubes, Ex vivo incubation, Blood, PBMC, RNA yield, RNA quality, Gene expression

## Abstract

**Background:**

Blood is a common source of RNA for gene expression studies. However, it is known to be vulnerable to pre-analytical variables. Although RNA stabilization systems have been shown to reduce such influence, traditional EDTA tubes are still widely used since they are less expensive and enable to study specific leukocyte populations. This study aimed to assess the influence of storage time and temperature between blood sampling and handling on RNA from peripheral blood mononuclear cells (PBMCs).

**Methods and results:**

Nine blood samples were collected in EDTA tubes from 10 healthy donors. One tube from each donor was immediately processed for PBMC isolation, while the others were first incubated at either 4 degrees Celsius (°C) or room temperature for 2, 4, 6 and 24 h. RNA yield and quality and the expression level of fourt housekeeping (B2M, CASC3, GAPDH, HPRT1) and 8 target genes (CD14, CD19, CD20, IL10, MxA, TNF, TNFAIP3, NR4A2) were compared between samples.

RNA yield, quality and integrity did not vary significantly with time and temperature. B2M was the most stable housekeeping gene, while the others were increasingly influenced by storing time, especially at 4 °C. Even when normalized to B2M, the expression level of some target genes, particularly TNFAIP3 and NR4A2, was highly affected by delays in blood processing at either temperature, already from 2 h.

**Conclusion:**

Pre-analytical processing has a great impact on transcript expression from blood collected in EDTA tubes, especially on genes related to inflammation. Standardized procedure of blood handling are needed to obtain reliable results.

**Supplementary Information:**

The online version contains supplementary material available at 10.1007/s11033-022-07320-5.

## Introduction

Advanced molecular biology techniques of gene expression profiling are gaining importance in research, diagnosis and prognosis of human diseases, as well as monitoring and prediction of drug therapies’ response, enabling the shift from a cohort-based to a personal-based approach. Such techniques rely on high quality sources of biomarkers that accurately reflect the pathophysiological status of the individual. Due to its minimally invasive method of collection, peripheral blood is a widely used RNA source. However, blood is highly susceptible to pre-analytical factors that overall affect downstream applications, such as sample collection methods, time and temperature conditions of pre-freezing transportation and storage, and time of post-freezing storage [1–11].

Ex vivo alteration of the mRNA transcript could be the main reason behind the high level of irreproducibility affecting biomedical research [12, 13]. Thus, more efforts are required to standardize pre-analytical procedures, particularly in light of the crucial role that biobanks are acquiring in research activities.

Multiple commercial kits have been developed to stabilize whole blood RNA immediately after phlebotomy [14, 15]. These methods are more expensive than traditional blood tubes, and depict the RNA profiles of all blood cell types, including granulocytes, erythrocytes, reticulocytes and platelets. Therefore, although they remove the influence of prolonged ex vivo incubation and provide a readily accessible source of RNA, they are not suitable for all purposes. Ethylenediaminetetraaceticacid (EDTA) blood collection tubes are therefore still widely used for gene expression studies on both whole and specific blood cell populations.

Standardized strategies and best practice protocols for blood collection and manipulation have been proposed to reduce pre-analytical variables [16–18]. International standard ISO 15189, which specifies requirements for quality and competence in medical laboratories, contains several sections focused on the pre-analytical phase [19]. The Horizon 2020 project SPIDIA4P aims to promote the standardization of pre-analytical workflows applied to personalized medicine by developing European and international standard documents and external quality assessment schemes [20]. However, to date, pre-analytical variability is still a crucial issue whose implications needs further investigation.

In this context, we designed a study to assess the influence of pre-analytical variables, namely storage time and temperature between blood sampling and handling, on RNA from peripheral blood mononuclear cells (PBMCs). In particular, we focused on evaluating changes in RNA quality and gene expression occurring after 2 h (h), 4 h, 6 h and 24 h delays in blood processing, at both 4 degrees Celsius (°C) and room temperature (RT). Our findings highlight the dramatic impact of pre-analytical processing on gene expression and emphasize the relevance of proper blood sample handling and the need of share standardized procedures to obtain reliable results.

## Methods

### Sample collection and experimental design

**Fig. 1:**
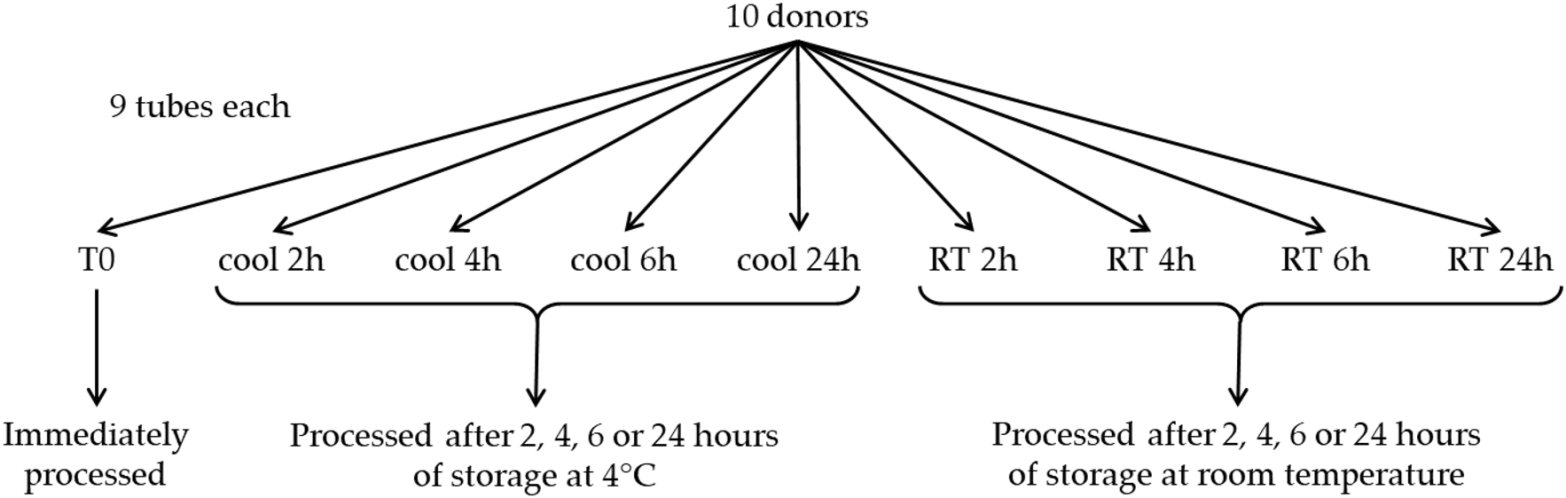
Graphical workflow

Whole blood samples were collected from 10 healthy subjects into 9 dipotassium ethylenediaminetetraaceticacid (K2-EDTA) Vacutainer™ tubes (6 ml blood per tube, Becton–Dickinson, Franklin Lakes, NJ, USA). The time course study was performed as follow: one tube from each participant was immediately processed for PBMC isolation (T0), the remaining tubes were stored for 2, 4, 6 or 24 h at either RT (i.e. 21 degrees Celsius) or 4 °C (Fig. 1). Two samples from one participant were lacking, corresponding to 6 h storage at RT and 4 °C, thus a total of 88 samples were collected. After this phase, PBMCs were isolated using Lymphoprep (Stemcell Technologies) according to standard techniques and stored at − 80 °C in RNA later (Invitrogen, Thermo Fisher Scientific) until RNA extraction.

Sample collected were stored in CRESM Biobank, a structured biobank for neurological and autoimmune diseases of the regional reference center for MS (CRESM), in Piedmont (Italy).

The study was approved by the Ethical committee of San Luigi Gonzaga University Hospital (approvals number 18390/2019). Written informed consent was obtained from each participant.

### RNA extraction and quality control

Total RNA was extracted using the semi-automated Maxwell 16 Rapid Sample Concentrator (RSC) instrument with Maxwell R 16LEV simply RNA Tissue Kit (Promega, Madison, USA), following the manufacturer’s instructions. Total RNA concentration was measured using Nanodrop® ND-1000 spectrophotometer (Celbio, Italy). RNA purity was evaluated by examining the optical density (OD) ratios 260/280 and 260/230. RNA integrity was assessed using Bioanalyzer (Eukaryote Total RNA Nano kit, Agilent, Thermo Fisher Scientific) and RNA integrity numbers (RIN) were calculated using the 2100 Expert software (Thermo Fisher).

### Real time PCR assay and data analysis

Total RNA was reverse-transcribed at a final concentration of 5 ng/μL using High-Capacity cDNA Reverse Transcription Kit (Thermo Fisher), according to the manufacturer’s protocol. A water sample was run as negative control of reverse-transcription. cDNA samples were stored at − 80 °C until use. Real-time PCR reactions were carried out in 96-well PCR plate on the ABI StepOne Plus real-time PCR System (Applied Biosystems, Monza, Italy), using Universal Master Mix (Life Technologies, Monza, Italy) and TaqMan® gene expression assays (Thermo Fisher). Transcript levels of 4 housekeeping genes and 8 target genes were assessed using the following probes: Beta-2-Microglobulin (B2M, Hs00187842_m1), CASC3 Exon Junction Complex Subunit (CASC3, Hs00201226_m1), Glyceraldehyde-3-Phosphate Dehydrogenase (GAPDH, Hs99999905_m1), Hypoxanthine Phosphoribosyltransferase 1 (HPRT1, Hs99999909_m1), CD14 Molecule (CD14, Hs02621496_s1), CD19 molecule (CD19, Hs00174333_m1), Membrane Spanning 4-Domains A1 (CD20, Hs00544818_m1MS4A1), Interleukin 10 (IL10, Hs00961619_m1), MX Dynamin Like GTPase 1 (MxA, Hs00182073_m1), Tumor Necrosis Factor (TNF, Hs00174128_m1), Tumor Necrosis Factor Alpha-Induced Protein 3 (TNFAIP3, Hs00234713_m1) and Nuclear Receptor Subfamily 4 Group A Member 2 (NR4A2, Hs00428691_m1). Real-time PCR reactions for each gene were performed in duplicate. A negative control of amplification was run in each amplification plate. Samples were subjected to incubation for 2 min at 50 °C followed by 10 min at 95 °C, then to 40 cycles of amplification at 95 °C for 15 s, and 60 °C for 60 s in the StepOne Real-Time PCR System (Life Technologies).

The threshold cycle (*Ct*) values were obtained analyzing each plate with SDS v2.3 software (Life Technologies). Mean values of duplicates with Standard Deviation (SD) lower or equal to 0.5 were accepted; if SD was higher than 0.5 the amplification reaction was repeated. Mean *Ct* values were analyzed by the comparative *Ct*-method. The expression level of candidate housekeeping genes was given as *Ct* value. The relative expression level of target genes normalized to each housekeeping gene was calculated applying the 2^−Δ*Ct*^ formula*,* where Δ*Ct* = (*Ct*_*target*_*-Ct*_*housekeeping*_*)*_*Time*_*x.* TimeX is any time point and storing temperature.

### Statistical analysis

The effect of storage time on RNA yield, quality and integrity and on gene expression levels was examined at each temperature. Differences between groups were evaluated by one-way repeated measures ANOVA followed by post hoc pairwise paired t-tests with Benjamini–Hochberg adjustment, or by Friedman’s test followed by Nemenyi’s post hoc test, as appropriate. Normality of distributions and sphericity assumption were checked by Shapiro’s and Mauchly’s tests, respectively. Mean changes in the Ct value of housekeeping genes were calculated as the mean of the differences between the Ct value of each individual at each time point and each temperature and the respective Ct value at T0. Mean percentage changes in the 2^−ΔCt^ value of target genes compared to the T0 value were calculated as the mean of the ratios of the difference between the 2^−ΔCt^ value at each time point and each temperature and the respective T0 value, relative to the T0 value, for each individual. P values < 0.05 were considered statistically significant. Analyses were performed with R version 4.0.2 (www.r-projet.org).

## Results

Nine blood samples from each of the 10 donors participating in the study were collected in EDTA tubes. One tube from each participant was immediately processed for PBMC isolation (T0), while the remaining tubes were stored at different conditions until processing (Fig. 1). A total of 88 RNA samples were obtained (the two 6 h time points from one participant were lacking). Statistically significant changes in RNA quality and gene expression levels were evaluated at two different temperatures (4 °C and RT) for storage times up to 6 h, which are the most likely real lab scenarios. Data on overnight storage were also shown for descriptive purposes.

### Effects of the pre-analytical processes on RNA yield, quality and integrity

**Fig. 2:**
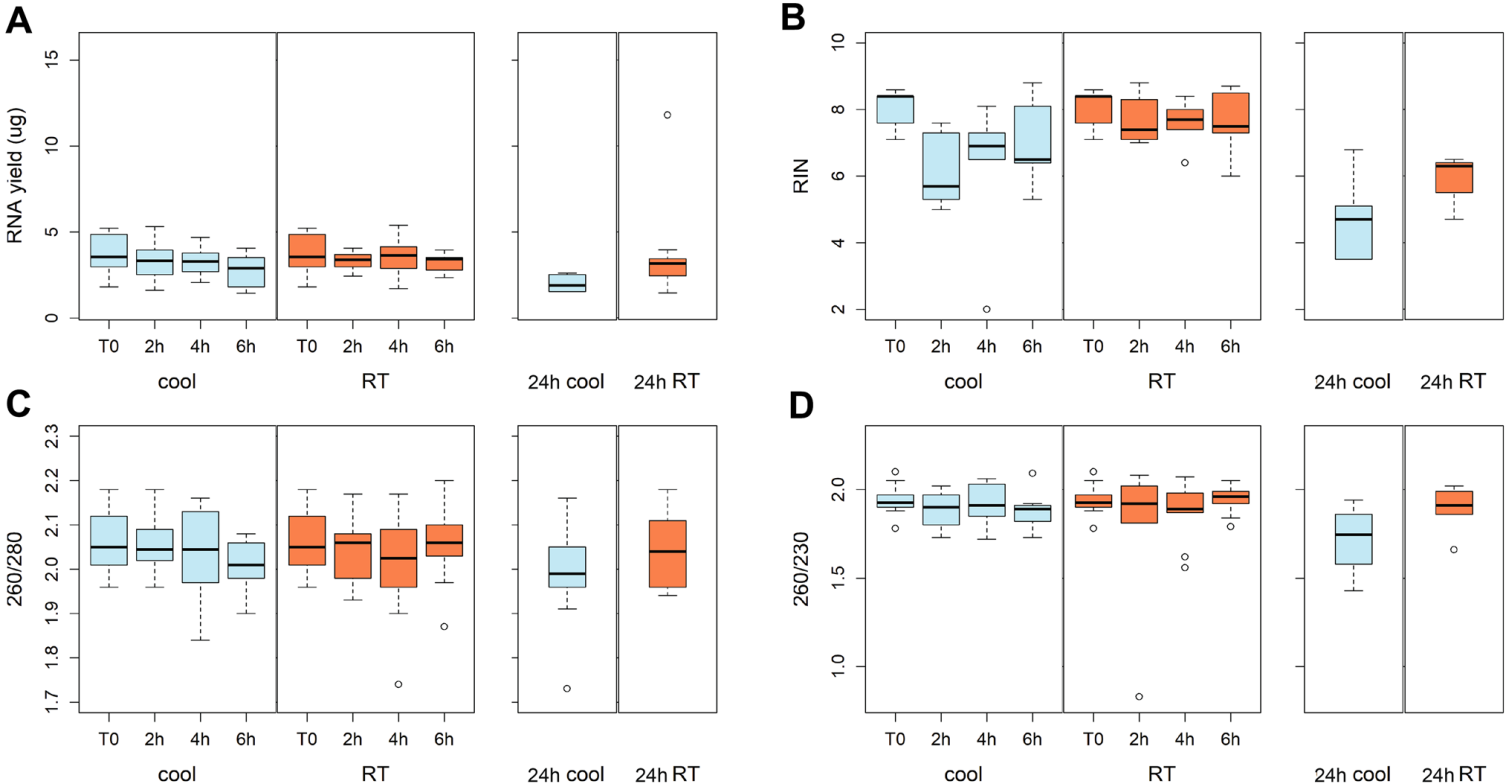
RNA yield (**A**), integrity (**B**) and purity, calculated as the ratio of the absorbance at 260 and 280 nm (**C**) and at 260 and 230 nm (**D**), measured at each temperature condition and storage time

RNA yield and purity were evaluated in all samples (n = 88), while the RNA integrity was evaluated in samples from 5 donors (n = 45). Incubation time up to 6 h did not significantly affect RNA characteristics at neither of the two storage temperature (repeated measures ANOVA, RNA yield, 4 °C: p = 0.08, RT: p = 0.43; 260/280, 4 °C: p = 0.56, RT: p = 0.63; 260/230, 4 °C: p = 0.59, RT: p = 0.66; RIN, 4 °C: p = 0.20, RT: p = 0.82) (Fig. 2). After 24 h incubation, the median RNA yield decreased from 3.55 to 1.89 and 3.15 µg at 4 °C and RT, respectively (Fig. 2A). Moreover, the RIN value of 3 samples stored at 4 °C and one sample stored at RT decreased below 5 (Fig. 2B), which is the standard cut-off for application in gene expression studies [21].

### Effects of the pre-analytical processes on housekeeping gene expression

**Fig. 3:**
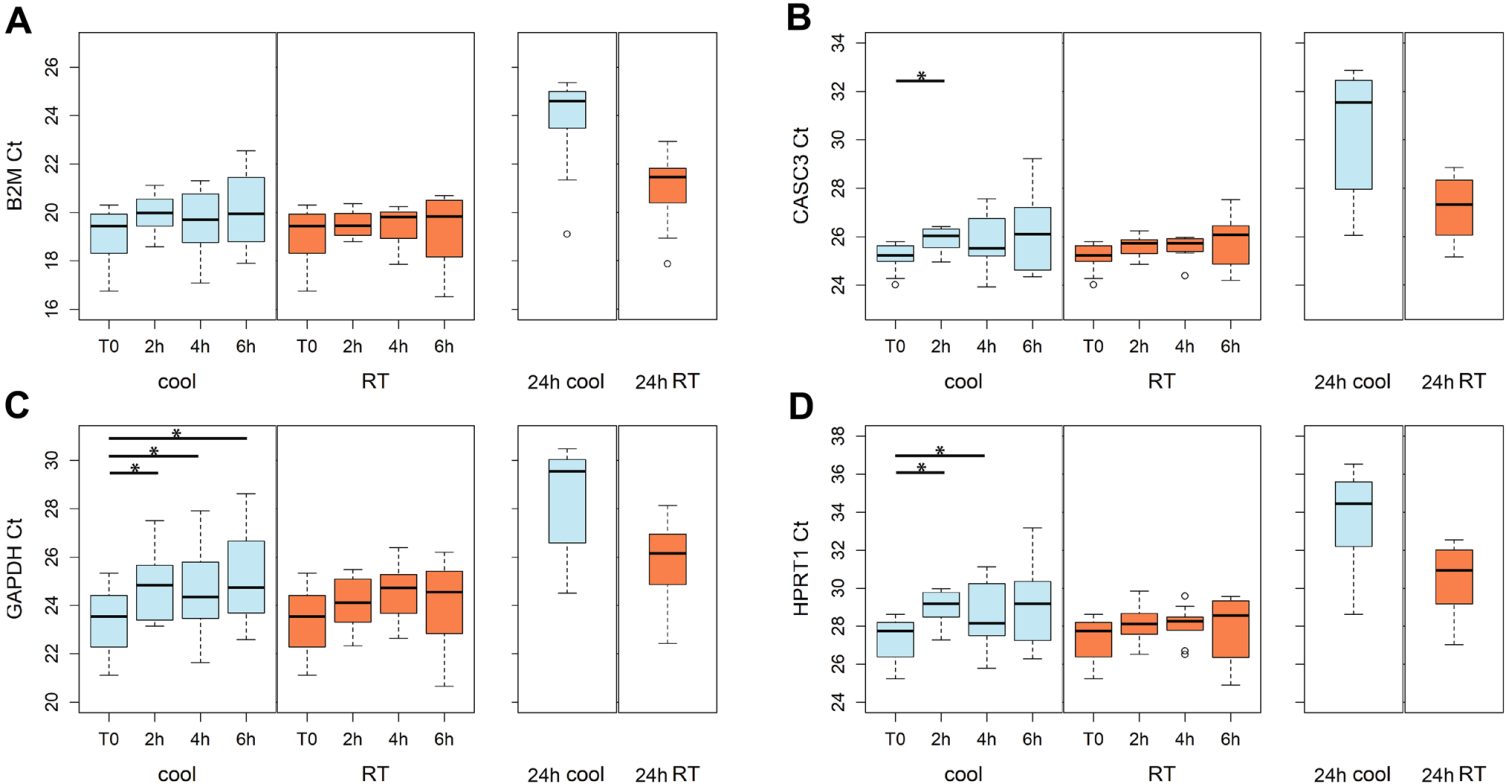
Gene expression level of four housekeeping genes: B2M (**A**), CASC3 (**B**), GAPDH (**C**) and HPRT1 (**D**). The Ct value was measured by real-time PCR at each temperature condition and storage time. * 0.01 ≤ p < 0.05

Four housekeeping genes (B2M, CASC3, GAPDH and HPRT1) were selected for the evaluations. Overall, their expression level showed a greater stability over time at RT (Fig. 3). On the other hand, significant changes in the Ct value were observed for all the genes analyzed, with the exception of B2M (Fig. 3A), when samples were stored at 4 °C (repeated measures ANOVA, B2M, 4 °C: p = 0.13, RT: p = 0.47; CASC3, 4 °C: p = 0.048, RT: p = 0.08; GAPDH, 4 °C: p = 0.03, RT: p = 0.08; HPRT1, 4 °C: p = 0.02, RT: p = 0.21). In particular, the expression level of CASC3, GAPDH and HPRT1 significantly decreased already at 2 h, as their Ct value increased (post hoc pairwise paired t-tests, T0 vs 2 h, CASC3: p = 0.02; GAPDH: p = 0.04; HPRT1: p = 0.01) (Fig. 3B–D). Mean changes in the Ct value of each gene at each storage time and temperature condition relative to T0 is summarize in Supplementary table 1. Notably, storage for 24 h was associated to great mean increases in the Ct value of the genes, of least 2 points at RT and 4.7 at 4 °C, which corresponds respectively to a reduction of about 75% and 96% in the original number of mRNA copies. Due to its stability over time, B2M was selected as the best housekeeping gene for subsequent analysis. On the contrary, HPRT1 was selected as the worst housekeeping gene since it was unstable and the least expressed of all the 4 genes.

### Effects of pre-analytical processes on normalized gene expression

**Fig. 4:**
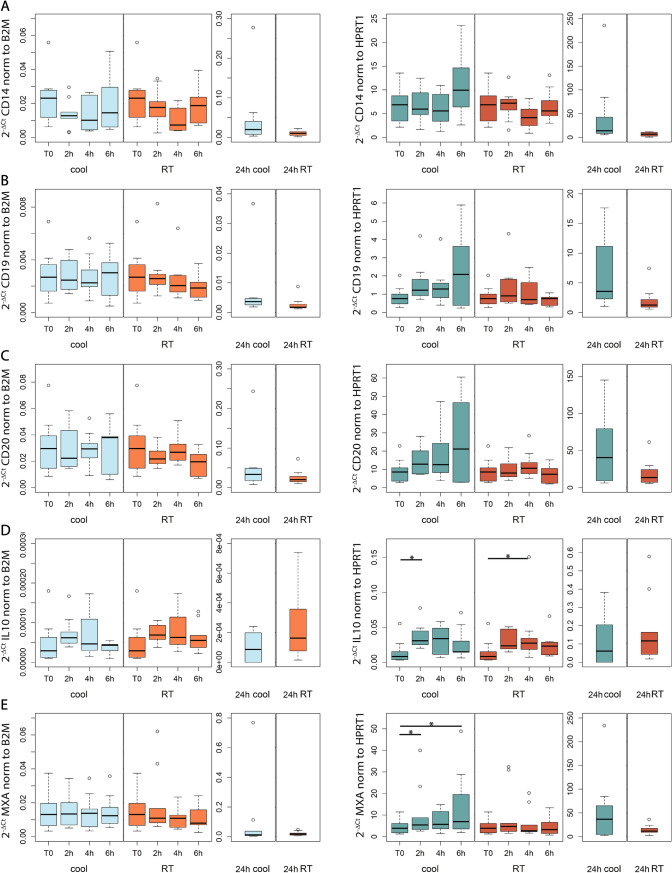

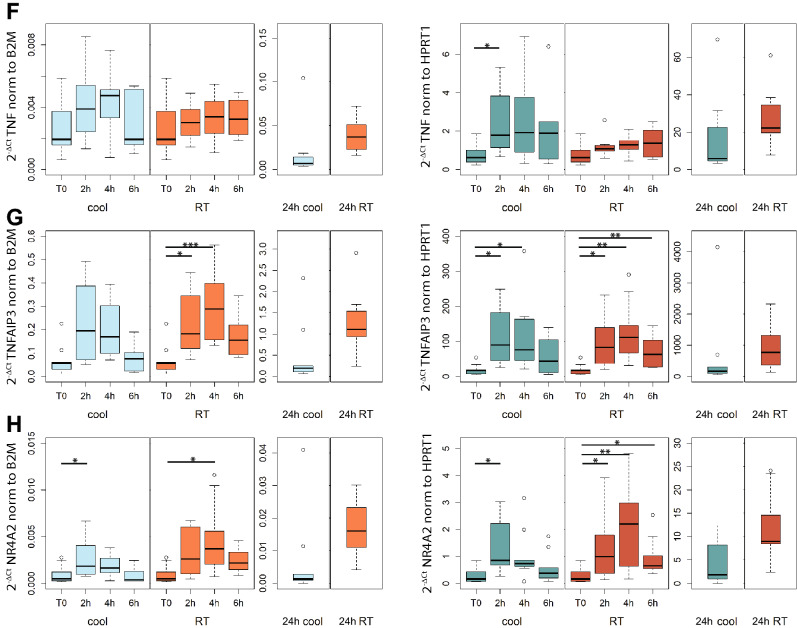
Gene expression levels of eight genes: CD14 (**A**), CD19 (**B**), CD20 (**C**), IL-10 (**D**), MXA (**E**), TNFα (**F**), TNFAIP3 (**G**) and NR4A2 (**H**). 2^−∆Ct^ values were calculated using B2M (left panels) or HPRT1 (right panels) as housekeeping gene, at each temperature condition and storage time. * 0.01 ≤ p < 0.05, ** 0.001 ≤ p < 0.01, ***p < 0.001

The effect of storage time and temperature on the expression level of 8 target genes (CD14, CD19, CD20, IL10, MxA, TNF, TNFAIP3 and NR4A2) was evaluated. They were selected as representative genes of a broad spectrum of molecular targets, including membrane proteins, nuclear receptors and cytokines, and because of their relation with multiple sclerosis [22–30]. The expression level of each target gene was normalized to the expression level of the housekeeping gene and expressed as 2^−ΔCt^ value (Fig. 4). When normalized to B2M, only the expression level of NR4A2 and TNFAIP3 showed significant changes over time. In particular, NR4A2 expression showed the highest increase after 2 h at 4 °C (repeated measures ANOVA followed by post hoc pairwise paired t-tests, p = 0.046) and after 4 h at RT (Friedman’s test followed by Nemenyi’s post hoc test, p = 0.01), while TNFAIP3 expression was significantly higher at 2 h and 4 h at RT (Friedman’s test followed by Nemenyi’s post hoc test, 2 h vs T0: p = 0.04; 4 h vs T0: p = 0.0008). When normalized to HPRT1, 5 out of 8 genes showed significant changes over time, especially at 4 °C. IL-10 expression increased at both 4 °C and RT (Friedman’s test followed by Nemenyi’s post hoc test, 4 °C, 2 h vs T0: p = 0.04; RT, 4 h vs T0: p = 0.01), as well as TNFAIP3 (Friedman’s test followed by Nemenyi’s post hoc test, 4 °C, 2 h vs T0: p = 0.01, 4 h vs T0: p = 0.04; RT, 2 h vs T0: p = 0.02, 4 h vs T0: p = 0.003, 6 h vs T0: p = 0.005) and NR4A2 (Friedman’s test followed by Nemenyi’s post hoc test, 4 °C, 2 h vs T0: p = 0.03; RT, 2 h vs T0: p = 0.03, 4 h vs T0: p = 0.008, 6 h vs T0: p = 0.01). On the other hand, the expression level of MxA (Friedman’s test followed by Nemenyi’s post hoc test, 4 °C, 2 h vs T0: p = 0.046, 4 h vs T0: p = 0.01) and TNF-α (Friedman’s test followed by Nemenyi’s post hoc test, 4 °C, 2 h vs T0: p = 0.02) significantly increased over time only when samples were stored at 4 °C. The mean percentage change in the 2^−ΔCt^ value of each gene at each time point compared to T0 is summarized in Supplementary table 2. Notably, storage for 24 h was associated to considerable changes in gene expression levels, particularly of genes related to inflammation, which showed increases of over 1000%.

## Discussion

Gene expression profiling of blood cells is taking hold in research, diagnosis and monitoring of many human diseases, especially those characterized by inflammation and activated immune responses. However, blood is known to be vulnerable to pre-analytical variables that may alter gene expression ex vivo [1–11]. Blood collection in RNA stabilization systems have been shown to successfully reduce the influence of pre-freezing storage conditions on RNA quality and gene expression [14, 15]. Nevertheless, traditional EDTA tubes are still widely used for clinical and research purposes, because they are less expensive and enable to study specific leukocyte populations.

Few studies have investigated the effect of bench times on RNA quality and transcript expression of blood cells collected in EDTA tubes [2–4, 31, 32], and none of them involved subsequent PBMC isolation. Here we evaluated the effect of different bench times, defined as the time between blood collection and the beginning of the procedures for PBMCs isolation. We focused on changes in RNA yield, purity, integrity and specific gene expression levels occurring after 2 to 6-h incubation of blood tubes at both 4 °C and RT, which are common conditions in laboratory routine. We also showed changes occurring after 24-h incubation, representing an extreme working condition.

According to most findings in the literature [2, 8, 31–34], we observed that incubation up to 6 h at both 4 °C and RT did not significantly affect RNA yield, purity and integrity. However, after 24 h the RIN value of some samples, even if stored at 4 °C, decreased below 5, which is considered the quality cut-off for gene expression studies [21]. This is in contrast with studies that describe RIN values above 5 even beyond 24-h incubations [2, 8, 31, 33], and may be due to additional degradation induced by the PBMC separation procedures. Notably, the RIN score is mainly based on the features of ribosomal RNA and does not represent a specific quality score for mRNA [35], which is the target for gene expression studies.

Besides acceptable levels of mRNA quality and integrity, a valid reference for normalizing gene expression results is required to avoid misinterpretations. We therefore evaluated over time variability of 4 candidate genes (B2M, CASC3, GAPDH and HPRT1) extensively used as housekeeping genes. Ct values altogether seemed to increase over time, but we surprisingly observed significant changes only when samples were stored at 4 °C, for all genes except B2M. Already after 2 h at 4 °C all genes reached a mean increase in the Ct value of about 1 point or more, which means that the number of transcripts had approximately halved. On the other hand, at RT the Ct mean increase over time was limited, particularly for B2M and CASC3. It should be noted that after 24 h at RT the most stable housekeeping genes showed a mean increase of 2 points in the Ct value. Assuming an optimal PCR efficiency, in which gene-specific amplicons double from cycle to cycle, this variation corresponds to a 75% decrease in the original number of mRNA transcripts. After 24 h at 4 °C, instead, even the most stable housekeeping gene showed a mean increase of about 5 points in the Ct value, corresponding to a 96% decrease in the original number of mRNA copies. Variations in the expression levels of housekeeping genes following ex vivo incubation have already been described in literature. Baechler and colleagues measured by real-time RT-PCR the expression levels of a group of genes, including B2M, from whole blood of 2 control individuals drawn into EDTA tubes and incubated at RT for 0, 2, 6, 12, 24, and 48 h [4]. Their results cannot be directly compared to ours due to differences in blood handling and because they normalized the B2M expression to that of EEF1A1. However, they observed notable changes in gene expression occurring as early as 2 h after blood draw [4]. On the other hand, 2 studies explored changes in the expression of GAPDH following 24 h incubation. Das and colleagues compared the expression level of GAPDH in neutrophils isolated from blood samples from 6 healthy donors collected in EDTA tubes and incubated at RT for 0, 1, 2 and 3 days [34]. They observed an increase over time in the number of copies per million cells, with a 5.8 fold change at day 1 compared to the day 0 value, apparently in contrast with our findings [34]. Differences in the cell type analyzed, in the cell isolation method and the use of an absolute instead of a relative method for gene expression quantification may explain such inconsistencies. Although in the opposite direction, however, this finding agrees in ascribing dramatic changes in the expression levels of housekeeping genes to delays in blood sample processing. Finally, Malentacchi and colleagues compared the expression level of GAPDH in whole blood samples from 1 donor collected in EDTA tubes and stored or transported at RT or 4 °C for 1 to 4 days [2]. Reported changes in the GAPDH expression over time at all storage conditions did not reach statistical significance after adjusting for multiple testing, but in light of the low sample size examined, the results of this study should as well focus attention on the serious consequences of long-term blood storage on the expression of commonly used reference genes.

Due to the exponential nature of the real-time PCR kinetic, even small changes in Ct values may have a large impact on gene expression calculation. We therefore selected B2M and HPRT1 as a model to compare gene expression results obtained by normalizing to a stable and an unstable reference. We analyzed the expression level of 8 target genes: CD14, CD19, CD20, IL10, MxA, TNF, TNFAIP3 and NR4A2. They represent a broad spectrum of molecular targets, including membrane proteins, nuclear receptors and cytokines, with different functions ranging from inflammation to anti-viral activities. They are also of particular interest in the neurological field since they are involved in the pathogenesis of multiple sclerosis and in the disease-specific treatment response [22–30]. When using an unstable reference such as HPRT1, we observed significant changes over time in 5 out of 8 genes: MxA, IL-10, TNF, TNFAIP3 and NR4A2. On the other hand, when using a stable reference such as B2M, we detected significant changes for TNFAIP3 and NR4A2 only. This is a clear example that using a suboptimal reference may lead to gene expression miscalculations and exalt meaningless variations or maybe conceal true biological differences. Storing temperature seemed to have instead only minor effects on variability of relative gene expression over time.

TNFAIP3 and NR4A2 belong to a class of transcription factors and transcription factor modulators with inhibitory activities on the pro-inflammatory molecule NF-kB [36, 37]. Compared to T0, the expression level of TNFAIP3 and NR4A2 showed a significant pick of increase after 2–4 h and then a mild decrease after 6 h of ex vivo incubation at both 4 °C and RT. This is in line with previous studies investigating the influence of short and long delays in blood processing on whole blood gene expression profiles [31, 32]. Transcriptomic profiles, especially of genes involved in apoptosis, stress signaling and DNA damage repair, were found to be highly affected by delays as little as 2 h before blood fractionation by Hebels and colleagues. The authors also provided a list of candidate markers of bench time effect, which includes TNFAIP3 [31]. In addition, Franken and colleagues observed that delays of less than 30 min affect the expression of genes with a prominent role for NF-kB-, glucocorticoid- and cancer-mediated networks [32]. TNFAIP3 and NR4A2 were also found differentially expressed in PBMCs after a 4 h delay when blood was collected in acid citrate dextrose tubes [38].

In our study, 24 h storage of blood samples at both 4 °C and RT was associated to dramatic changes in gene expression levels, particularly of genes involved in inflammatory processes such as TNF, TNFAIP3 and NR4A2 which displayed a considerable increase. This is not surprising, considering that changes in the environment of blood cells after phlebotomy could trigger stress signals and inflammatory processes [39]. In contrast, Shen and colleagues suggested that ongoing cellular processes have a negligible effect and blood samples stored at 4 °C for 1–7 days are suitable for RNA sequencing analysis, since they found only few differentially expressed genes compared to samples stored at 4 °C for 4 h [8]. On the other hand, Romero and colleagues indicated that RNA-sequencing output is drastically affected by RNA degradation, but in contexts where RIN and the outcome of interest are not associated, its effect could be controlled using a linear model framework [33].

Overall, our findings showed that specific mRNA transcripts from blood samples collected in traditional tubes such as EDTA tubes are differently affected by delays in blood processing as short as 2 h, even when stored at low temperature. While inflammation-related genes increase their expression over time, housekeeping genes that are commonly used as reference genes decreased, with consequences on relative gene expression results. This phenomenon has clear implications in research and monitoring of autoimmune and inflammatory disease and in biobanking in general. Increasing evidences support the need of standardized pre-analytical procedures to guarantee reproducible results, however it is difficult to achieve as samples are often collected at different times during the day and then processed in a single working session. Whenever possible, collecting blood in RNA stabilization systems could be a valid solution to this issue; time-course studies to evaluate the stability of reference and target genes of interest are crucial otherwise.

## Supplementary Information

Below is the link to the electronic supplementary material.

Supplementary file1 (DOCX 17 kb)

Supplementary file2 (DOCX 16 kb)
