# Supplementary material for: The impact of pre-freezing storage time and temperature on gene expression of blood collected in EDTA tubes
Source: Mol Biol Rep. 2022 Mar 12;49(6):4709–18. doi: 10.1007/s11033-022-07320-5 (PMC9262796; doi:10.1007/s11033-022-07320-5)
Supplement: Supplementary file 1 — Supplementary file1 (DOCX 17 kb) [file 11033_2022_7320_MOESM1_ESM.docx]

**Supplementary table 1**. Mean changes in the Ct value of housekeeping genes at each storage time and temperature condition compared to T0.

|  | **4°C** | | | | **RT** | | | |
| --- | --- | --- | --- | --- | --- | --- | --- | --- |
|  | **B2M** | **CASC3** | **GAPDH** | **HPRT1** | **B2M** | **CASC3** | **GAPDH** | **HPRT1** |
| 2h | 0.9 | 0.8 | 1.5 | 1.7 | 0.5 | 0.5 | 0.8 | 0.9 |
| 4h | 0.7 | 0.7 | 1.2 | 1.4 | 0.5 | 0.4 | 1.2 | 0.9 |
| 6h | 1.0 | 1.2 | 1.9 | 2.1 | 0.2 | 0.6 | 0.7 | 0.5 |
| ON | 4.7 | 5.3 | 5.2 | 6.5 | 2.0 | 2.0 | 2.5 | 3.1 |
