# Supplementary material for: The impact of pre-freezing storage time and temperature on gene expression of blood collected in EDTA tubes
Source: Mol Biol Rep. 2022 Mar 12;49(6):4709–18. doi: 10.1007/s11033-022-07320-5 (PMC9262796; doi:10.1007/s11033-022-07320-5)
Supplement: Supplementary file 2 — Supplementary file2 (DOCX 16 kb) [file 11033_2022_7320_MOESM2_ESM.docx]

**Supplementary table 2**. Mean percentage changes in the 2^-ΔCt^ value of each gene at each time point relative to T0.

|  | **B2M** | | | | | | | | **HPRT1** | | | | | | | |
| --- | --- | --- | --- | --- | --- | --- | --- | --- | --- | --- | --- | --- | --- | --- | --- | --- |
|  | **4°C** | | | | **RT** | | | | **4°C** | | | | **RT** | | | |
|  | 2h | 4h | 6h | ON | 2h | 4h | 6h | ON | 2h | 4h | 6h | ON | 2h | 4h | 6h | ON |
| CD14 | -3 | -21 | 48 | 82 | 22 | -37 | 12 | -46 | 52 | 10 | 146 | 571 | 42 | -28 | 31 | 28 |
| CD19 | 31 | 8 | 47 | 247 | 57 | -2 | -21 | 13 | 163 | 82 | 283 | 890 | 153 | 43 | -4 | 139 |
| CD20 | 30 | 18 | 50 | 202 | 35 | 30 | -29 | -6 | 156 | 109 | 322 | 832 | 107 | 86 | -7 | 107 |
| IL10 | 180 | 165 | 43 | 328 | 234 | 211 | 128 | 687 | 413 | 310 | 224 | 1296 | 384 | 295 | 166 | 1719 |
| MXA | 17 | 2 | 16 | 299 | 27 | -12 | -21 | 55 | 117 | 68 | 185 | 976 | 88 | 16 | 4 | 245 |
| TNF | 116 | 90 | 32 | 1047 | 61 | 52 | 60 | 1867 | 333 | 241 | 218 | 3143 | 135 | 105 | 104 | 3975 |
| TNFAIP3 | 476 | 317 | 202 | 1367 | 578 | 535 | 276 | 2285 | 980 | 616 | 669 | 4395 | 939 | 768 | 362 | 5516 |
| NR4A2 | 549 | 304 | 171 | 1960 | 842 | 1011 | 491 | 3615 | 1122 | 496 | 550 | 3501 | 1261 | 1272 | 533 | 8794 |
